# Profiling of Omega-Polyunsaturated Fatty Acids and Their Oxidized Products in Salmon after Different Cooking Methods

**DOI:** 10.3390/antiox7080096

**Published:** 2018-07-24

**Authors:** Kin Sum Leung, Jean-Marie Galano, Thierry Durand, Jetty Chung-Yung Lee

**Affiliations:** 1 School of Biological Sciences, The University of Hong Kong, Pokfulam Road, Hong Kong, China; sam612@connect.hku.hk; 2 Institut des Biomolécules Max Mousseron (IBMM), UMR 5247, CNRS Université de Montpellier, ENSCM, F-34093 Montpellier, France; jgalano@univ-montp1.fr (J.-M.G.); thierry.durand@umontpellier.fr (T.D.)

**Keywords:** salmon, cooking, lipid peroxidation, polyunsaturated fatty acids, neuroprostanes

## Abstract

Consumption of food containing n-3 PUFAs, namely EPA and DHA, are known to benefit health and protect against chronic diseases. Both are richly found in marine-based food such as fatty fish and seafood that are commonly cooked prior to consumption. However, the elevated temperature during cooking potentially degrades the EPA and DHA through oxidation. To understand the changes during different cooking methods, lipid profiles of raw, boiled, pan-fried and baked salmon were determined by LC-MS/MS. Our results showed that pan-frying and baking elevated the concentration of peroxides in salmon, whereas only pan-frying increased the MDA concentration, indicating it to be the most severe procedure to cause oxidation among the cooking methods. Pan-frying augmented oxidized products of n-3 and n-6 PUFAs, while only those of n-3 PUFA were elevated in baked salmon. Notably, pan-frying and baking increased bioactive oxidized n-3 PUFA products, in particular F-_4t_-neuroprostanes derived from DHA. The results of this study provided a new insight into the application of heat and its effect on PUFAs and the release of its oxidized products in salmon.

## 1. Introduction

Long chain n-3 polyunsaturated fatty acids (PUFA), including α-linolenic acid (ALA), eicosapentaenoic acid (EPA) and docosahexaenoic acid (DHA), are important to human health, as they have been reported to protect against cardiovascular disease and neurodegeneration and to reduce inflammation [1,2,3,4,5,6]. Consumption of dietary n-3 PUFAs is necessary, as ALA cannot be synthesized in the human body due to the absence of Δ^12^- and Δ^15^-desaturase enzymes and the low conversion efficiency of ALA to EPA and DHA in the metabolism. Both EPA and DHA are obtained through our diet, mainly from marine-based food such as fish oil, fatty fish and seafood [7]. However, due to hygiene reasons, it is rare to consume fish raw, and instead, it is commonly cooked by steaming, boiling, pan-frying, baking or roasting prior to consumption. The high temperature generated from these cooking methods could degrade EPA and DHA by breaking down the double bonds for oxidation. Some studies found that the levels of EPA and DHA in marine species were reduced after long-term storage and heat treatment [8,9,10,11], while others reported that the levels of EPA and DHA in certain fish species remained unchanged after cooking [12,13,14]. 

The high temperature generated during cooking increases the formation of free radicals and reactive oxygen species (ROS) and primarily releases products such as peroxides. On further oxidation, secondary products, namely the aldehydes or alcohol-derived compounds of the hydroperoxide primary metabolites, are released. For instance, F_2_-isoprostanes derived from arachidonic (AA) is a well-known biomarker for oxidative stress in diseases [15], but little is known about its role or generation in food. Likewise, an increasing amount of studies has shown that oxidized products formed through peroxidation of EPA and DHA are beneficial to our health. For example, F_3_-isoprostanes (F_3_-IsoPs) derived from EPA are anti-inflammatory and anti-thrombotic, as they lower the production of pro-inflammatory prostaglandins and thromboxanes derived from arachidonic acid (AA) by competing for the cyclooxygenase enzyme activity [16]. In addition, some neuroprostanes derived from DHA such as 4-(*RS*)-4-F_4_- neuroprostane (NeuroP) are cardio-protective by reducing the risk of ischemia injury in the heart. Furthermore, 4-(*RS*)-4-F_4_-NeuroP was found to prevent post-translational modification of the type 2 ryanodine receptor (RyR2) in cardiac cells [17]. Such an effect restored calcium homeostasis in the heart and reduced arrhythmia. A_4_-NeuroP and J_4_-NeuroP are proposed to be have anti-inflammatory properties [17,18] that could bind and activate peroxisome proliferator-activated receptors (PPARs) to reduce the expression of cytokines IL-6 and TNF-α in response to the inflammation of macrophages [19].

Nevertheless, oxidation of DHA also releases other oxidized products such as 4-hydroxy-2-hexenal (4-HHE). It is the major secondary lipid peroxidation products of DHA that is claimed to be neurotoxic in neuronal cells by augmenting ROS activity and down-regulating antioxidant enzyme and glutathione (GSH) levels [20]. However, recently, there have been studies suggesting that low dose 4-HHE is cardioprotective through the activation of Nrf2 in the vascular cells [21,22].

In this study, the effect of cooking methods on the quality of the PUFA of fatty fish (salmon) was investigated by examining the level of lipid peroxidation and the quantification of PUFA and the oxidized product content of the raw and cooked salmons.

## 2. Materials and Methods 

### 2.1. Fish Samples and Heat Treatment

Fresh, deskinned salmon filets were purchased from a local food market and stored in refrigerators (<4 °C) for 2 h before cooking and extraction for analysis. A total of 16 filets, each 100 g, was divided into four groups including raw as the control, boiling, pan-frying and oven-baking. Details of the cooking process are outlined below.

1. Boiling: Salmon filets were boiled individually on a heat plate in 800 mL of water initially at 99 ± 1 °C for 10 min.
2. Pan-frying: Salmon filets were fried on a medium-sized frying pan on a heat plate to a center temperature of 200 ± 10 °C for 10 min and were flipped over half way (5 min) through cooking.
3. Oven-baking: The oven was pre-heated to 200 °C. Salmon filets were individually placed on an aluminum foil-coated metal tray and baked at 200 °C for 15 min.

No cooking oil was added in any cooking method to ensure that the lipids of the samples were from the salmon only. Thereafter, the cooked salmons were cooled to room temperature and stored at −80 °C until analysis.

### 2.2. Extraction of Crude Oil 

Raw and cooked salmon samples (50 g) were chopped finely by hand. The oil content was derived in a Soxhlet extractor with 500 mL of n-hexane/diethyl ether (80:20, *v*/*v*) for 8 h. The oil collected was cooled to room temperature and dried completely using nitrogen gas. The dried oil was purged with nitrogen and stored at −80 °C until further analysis.

### 2.3. Measurement of Lipid Peroxidation

A portion of the oil sample (1 g) collected was used for the peroxide value (PV) test to determine total lipid peroxides and the primary lipid peroxidation products, and another portion (1 g) of the oil sample extracted was used to estimate malondialdehyde (MDA) content using the thiobarbituric acid reactive substances (TBARS) test.

The peroxide levels, expressed as peroxide value of the oil, were measured by the conversion of potassium iodide to release iodine according to the Toru et al. method [23]. In brief, 1.0 g of the oil sample was weighed in an Erlenmeyer flask (125 mL) and dissolved with 30 mL acetic acid/ chloroform (3:2, *v*/*v*) solution. A volume of 0.5 mL saturated potassium iodide solution was then added to the dissolved oil, and the mixture was diluted with 30 mL of distilled water. The sample mixture was first titrated with 0.01 M sodium thiosulfate until it turned pale yellow. Then, 1 mL of starch indicator was added, and titration was continued until the disappearance of the dark blue color in the solution. 

TBARS test was measured based on the reaction between TBA with MDA and other confounding components according to the Papastergiadis et al. method [24]. In brief, 0.5 g of oil were dissolved in 10 mL toluene and reacted with 10 mL TBA reagent. The reaction mixture was swirled and vigorously shaken for 4 min, and the layers were separated by a separatory funnel. The lower phase was collected and heated in a boiling water bath for 10 min. After cooling down under running tap water, the absorbance of the samples was measured by spectrophotometer at 530 nm, and the concentration of MDA was calculated by using the equation generated by the MDA standard curve.

Corn oil (Lion & Globe, Hong Kong, China) purchased from a local market was stored at room temperature for 4 years to serve as the positive control for the PV and TBARS tests.

### 2.4. Extraction of Polyunsaturated Fatty Acids and Oxidized Products

PUFA and oxidized lipid products were extracted from salmon samples by Folch extraction as reported previously [25]. In brief, 0.05 g of finely-chopped salmon samples were homogenized in 10 mL of Folch solution (chloroform/methanol 2:1 *v*/*v* + 0.05% BHT) using a blade homogenizer (T25, ULTRA-TURRAX, IKA, Guangzhou, China). Afterwards, 2 mL of 0.9% NaCl were added to create a phase separation and extraction. After centrifugation at 800× *g* for 10 min at 4 °C, the lower organic phase was transferred to a glass vial, and the solvent was evaporated under a stream of nitrogen gas. The dried extract was re-suspended in 1 M potassium hydroxide in methanol (1:1). The samples were then hydrolyzed overnight, in the dark, at room temperature (25 °C). After hydrolysis, the samples were cooled and neutralized by hydrochloric acid. Finally, formic acid (pH 4.5) was added, mixed together with heavy isotope internal standards 4-(*RS*)-4-F_4t_-NeuroP-d_4_ and 10-F_4t_-NeuroP-d_4_ synthesized by Institut des Biomolécules Max Mousseron (IBMM, Montpellier, France) and isoprostanes (IsoP) including 5-F_2t_-IsoP-d_11_, 15-F_2t_-IsoP-d_4_, PGF_2α_-d_4_, hydroxyeicosatetraenoic acid (HETE), namely 5(*S*)-HETE-d_8_, 12(*S*)-HETE-d_8_, 15(*S*)-HETE-d_8_, 20-HETE-d_6_, AA-d_8_, EPA-d_5_ and DHA-d_5_ (Cayman Chemicals, Ann Arbor, MI, USA). Finally, the samples were cleaned and extracted using anionic exchange solid phase extraction columns (60 mg, Oasis MAX, Waters, Milford, MA, USA), as described in our previous study [26].

### 2.5. Quantification of Polyunsaturated Fatty Acids and Its Oxidized Products 

**Figure 1:**
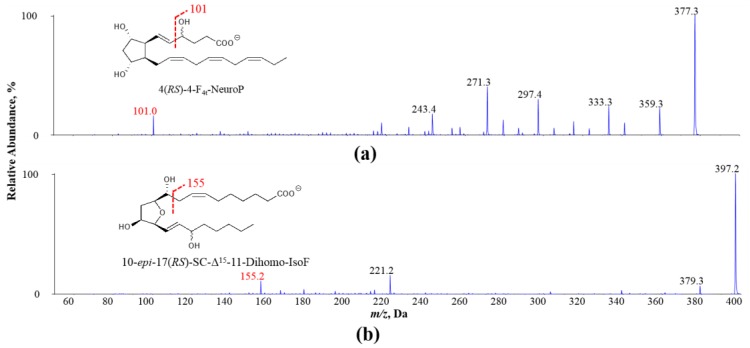
Exemplary MS/MS spectra of oxidized PUFA products (**a**) 4(*RS)*-4-F_4t_-NeuroP derived from DHA and (**b**) 10-*epi*-17(*RS*)-SC-Δ^15^-11-dihomo-IsoF derived from AdA: adrenic acid; NeuroP: neuroprostane; IsoF: isofuran.

The extracted PUFA and its oxidized products were analyzed according to previous studies with modifications [27,28]. A liquid chromatography tandem mass spectrometry (LC-MS/MS) system consisting of a 1290 Infinity LC system (Agilent, Santa Clara, CA, USA) with a C18 column (2.6 µm particle size, 150 × 2.1 mm, Phenomenex, Torrance, CA, USA) set to 30 °C was used. The mobile phase consisted of 0.1% formic acid in water (A) and 0.1% formic acid in acetonitrile (B). The flow rate was set to 200 µL/min, and the injection volume was 10 µL. The gradient was first maintained at 10% B from 0 to 1 min, then a linear gradient from 10% B to 98% B for 7 min, then 98% B held for 4 min and, finally, a linear gradient from 98% B to 10% B for 0.1 min. Thereafter, the column was re-equilibrated to the starting condition. A QTrap 3200 triple quadrupole mass spectrometer (Sciex Applied Biosystems, Framingham, MA, USA) coupled to the LC was operated at negative atmospheric pressure chemical ionization (APCI) mode. The spray voltage was set to −4000 V, and nitrogen gas was used as the curtain gas. The scan mode was multiple reaction monitoring (MRM), and the MS/MS transition (Figure 1) was monitored according to our previous reports [27,28]. Quantitation of each analyte was determined by relating the peak area with its corresponding deuterated internal standard peak including AA, EPA, DHA, 5(*S*)-, 12(*S*)-, 15(*S*)-, 20(*S*)-HETE, 15-F_2t_-IsoP, 5-F_2t_-IsoP, 4-(*RS*)-4-F_4t_-NeuroP and 10-F_4t_-NeuroP. For the analytes without the corresponding deuterated internal standards, i.e., adrenic acid (AdA), 8(*S*)-, 9(*S*)-, 11(*S*)-HETE, 7-F_2t_-dihomo-IsoP, 17-F_2t_-dihomo-IsoP, 7-F_2t_-dihomo-isofuran, 17-F_2t_-dihomo-isofuran, resolvin E1, 5-F_3t_-IsoP, 8-F_3t_-IsoP, 15-F_3t_-IsoP, resolvin D1, 4(*RS*)-ST-Δ^5^-8-neurofuran, 14(*RS*)-14-F_3t_-IsoP and 4-F_3t_-IsoP, quantitation was done by using deuterated internal standards with the relative response factor.

### 2.6. Extraction of Reactive Aldehydes, 4-Hydroxy-2(E)-Hexenal and 4-Hydroxy-2-Nonenal

Reactive aldehydes including 4-HHE and 4-HNE were extracted and derivatized from salmon samples according to Douny et al., with modifications [29]. In brief, 1 g of salmon samples was homogenized with 200 μL of BHT solution (1 mg/mL in ethanol) and 2200 μL of ethanol-water (50:50 *v*/*v*) by using a blade homogenizer (T25, ULTRA-TURRAX, IKA). A volume of 100 μL of internal standard mix containing 4-HHE-d_3_ and 4-HNE-d_3_ (0.25 ng/μL in ethanol) was added to the homogenates and vortexed for 1 min. Afterwards, the samples were centrifuged at 2100× *g* for 10 min. A volume of 2 mL supernatant was collected, filtered with hydrophilic filter and transferred into a new glass tube. Then, 2 mL 0.05 M 2,4-dinitrophenylhydrazine (DNPH)solution in acetonitrile/acetic acid (9:1 *v*/*v*) were added to the extract, and the samples were incubated in a 60 °C water bath for 2 h to derivatize the reactive aldehydes and internal standards. Thereafter, 2 mL of Milli-Q water and 2 mL of hexane were added to the mixture. To increase the yield, the derivatized extract was collected again with 2 mL hexane. The hexane extracts were combined and dried under a stream of nitrogen. The dried extract was resuspended with 100 μL of 0.1% acetic acid/acetonitrile (60:40 *v*/*v*), and LC-MS/MS analysis was performed immediately.

### 2.7. Quantification of 4-HHE and 4-HNE 

4-HHE and 4-HNE were measured in the samples by LC-MS/MS using ExionLC™ AC analytical HPLC with a C18 column (2.6 µm particle size, 150 × 2.1 mm, Phenomenex, Torrance, CA, USA) maintained at 30 °C. The mobile phase consisted of 0.1% acetic acid in water (A) and 0.1% acetic acid in acetonitrile (B). The flow rate was set to 300 µL/min, and the injection volume was 20 µl. The gradient was first increased from 40% B to 65% B for 8.5 min, then gradually increased to 100% B for 4 min and maintained for 7.5 min. Thereafter, the column was re-equilibrated to the starting condition. A Sciex X500R QTOF System mass spectrometer (Sciex Applied Biosystems, Framingham, MA, USA) coupled to the LC was operated at negative electrospray ionization, where the source temperature was set to 500 °C. The scan mode was multiple reaction monitoring (MRM), and the MS/MS transition was monitored as 293.09→167.01 for 4-HHE-DNPH, 335.14→167.01 for 4-HNE-DNPH, 296.09→167.01 for 4-HHE-d_3_ and 338.14→167.01 for 4-HNE-d_3_. Quantitation of 4-HHE and 4-HNE was determined by relating the peak area to its corresponding deuterated internal standard peak.

### 2.8. Statistical Analysis

Statistical analysis was performed using GraphPad Prism Version 6.0 (GraphPad Prism, La Jolla, CA, USA). All values were expressed as the mean ± standard deviation (SD). Differences between more than three groups were analyzed by 1-way analysis of variance (ANOVA), and *p* < 0.05 was noted as statistically significant.

## 3. Results

### 3.1. Different Cooking Methods Did Not Reduce PUFAs’ Levels in Salmon

**Figure 2:**
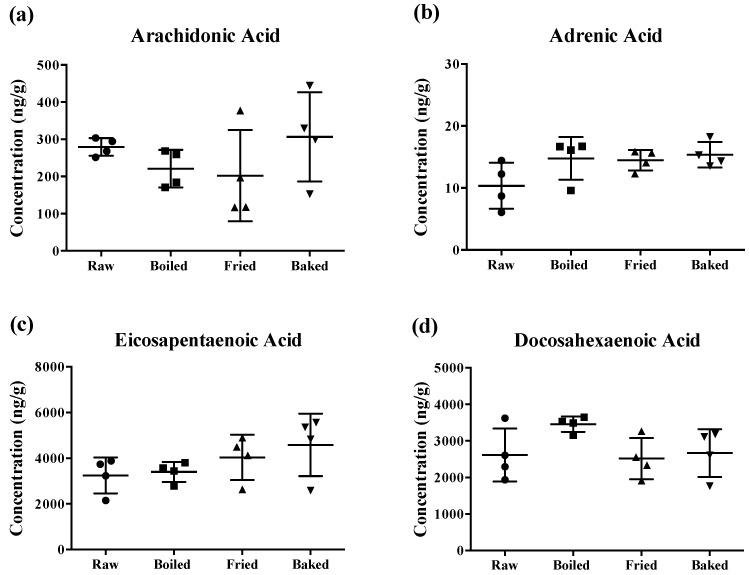
Concentration of polyunsaturated fatty acids measured in salmon samples. Graphs represent (**a**) arachidonic acid, (**b**) adrenic acid, (**c**) eicosapentaenoic acid and (**d**) docosahexaenoic acid measured in salmon samples. Data are presented as the mean ± S.D. (*n* = 4). Raw: control salmons without any cooking; boiled: salmons cooked by boiling; fried: salmons cooked by pan-frying; baked: salmons cooked by oven-baking.

The content of PUFAs determined in the prepared salmon samples is shown in Figure 2. Surprisingly, different cooking methods did not significantly reduce the concentration of AA, AdA, EPA and DHA of the salmons. Although there was a tendency for a significant change in some PUFA levels by the heat treatments, the large standard deviation of the mean (~50%) hampered the statistical relevance. Notably, the large deviation was more prominent when the salmon was fried or baked.

### 3.2. Elevated Lipid Peroxidation Was Found in Pan-Fried and Oven-Baked Salmons

**Figure 3:**
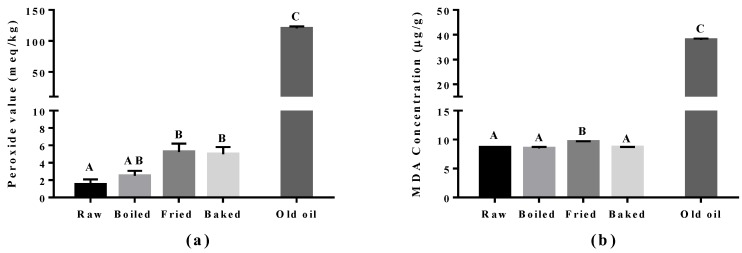
Level of lipid peroxidation in salmon samples measured by the peroxide value (PV) test and the thiobarbituric acid (TBARS) test. Graphs showing (**a**) PV and (**b**) malondialdehyde (MDA) measured in the oil extracted from salmon samples. Data are presented as the mean ± S.D. (*n* = 4). Raw: control salmons without any cooking; boiled: salmons cooked by boiling; fried: salmons cooked by pan-frying; baked: salmons cooked by oven-baking; old oil: corn oil expired for four years as the positive control. A similar letter denotes no statistical differences between samples, otherwise it is statistically significant at *p* < 0.05.

Lipid peroxidation was assessed in the cooked salmon and in old corn oil by the PV and TBARS tests (Figure 3). The corn oil (expired for four years) was used as a positive control and had approximately a 60-fold higher level for the PV value and a five-fold higher MDA level compared to raw salmon. Boiling did not increase PV and MDA significantly compared to raw salmon. However, a significant increase in PV was found in pan-fried and oven-baked salmon, and increased MDA was found in pan-fried salmon only, compared to other salmon samples. The elevation of peroxides showed that pan-frying and oven-baking indeed increased the extent of primary oxidation in the salmon. In addition, the higher level of MDA observed in pan-fried salmon suggested it to be more detrimental compared to other cooking methods.

### 3.3. Elevated 4-HHE and 4-HNE Were Found in Cooked Salmons

**Figure 4:**
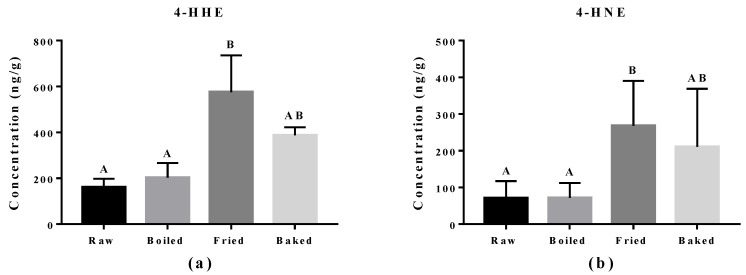
Levels of 4-hydroxy-2(E)-hexenal (4-HHE) and 4-hydroxy-2-nonenal (4-HNE) in salmon samples measured by LC-MS/MS. Graphs showing (**a**) 4-HHE and (**b**) 4-HNE in salmon samples. Data are presented as the mean ± S.D. (*n* = 6). Raw: control salmons without any cooking; boiled: salmons cooked by boiling; fried: salmons cooked by pan-frying; baked: salmons cooked by oven-baking. Similar letters denote no statistical differences between samples, otherwise it is statistically significant at *p* < 0.05.

Reactive aldehydes derived from n-3 PUFA and n-6 PUFA, namely 4-HHE and 4-HNE respectively, were measured in the salmon samples by LC-MS/MS (Figure 4). A significant elevation of 4-HHE and 4-HNE was found in pan-fried samples compared to raw and boiled salmon samples. Similar to what was observed in the PV and TBARS tests, pan-fried salmon contained the highest levels of 4-HHE and 4-HNE, followed by oven-baked, boiled and raw salmons. Among all salmon samples, the level of 4-HHE was higher compared to the level of 4-HNE likely due to higher n-3 PUFA than n-6 PUFA content in the salmons. The elevation of 4-HHE and 4-HNE found in pan-fried salmons indicated that lipid peroxidation of both n-3 PUFA and n-6 PUFA was escalated by pan-frying.

### 3.4. Elevated Oxidized PUFA Products Were Found in Cooked Salmons

Oxidized PUFA products released by enzymatic and non-enzymatic oxidation of the salmon samples were measured by using LC-MS/MS. A total of 25 out of 42 were successfully detected. No cooking methods showed much effect on the level of enzymatic-oxidized PUFA products of the salmon. Only two types of analytes, RvE1 enzymatically derived from EPA, after pan-frying, and 14-hydroxy-DHA (HDHA) derived from DHA by both enzymatic and non-enzymatic oxidation, after oven-baking, were elevated significantly (Table 1). 

**Figure 5:**
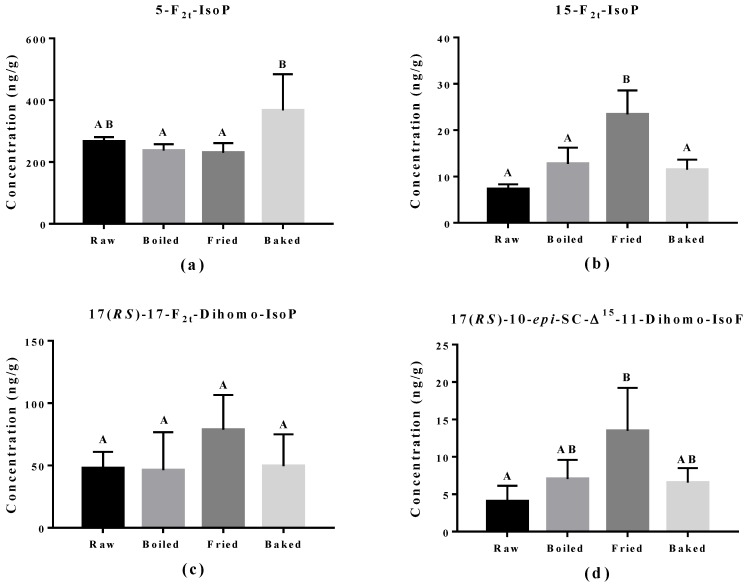
Concentration of non-enzymatically oxidized n-6 PUFA products measured in salmon samples. Graphs represent (**a**,**b**) F_2_-IsoPs derived from arachidonic acid and (**c**,**d**) dihomo-IsoP and dihomo-isofuran derived from adrenic acid. Data are presented as the mean ± S.D. (*n* = 4). Raw: control salmons without any cooking; boiled: salmons cooked by boiling; fried: salmons cooked by pan-frying; baked: salmons cooked by oven-baking. Similar letters denote no statistical differences between samples, otherwise it is statistically significant at *p* < 0.05.

**Figure 6:**
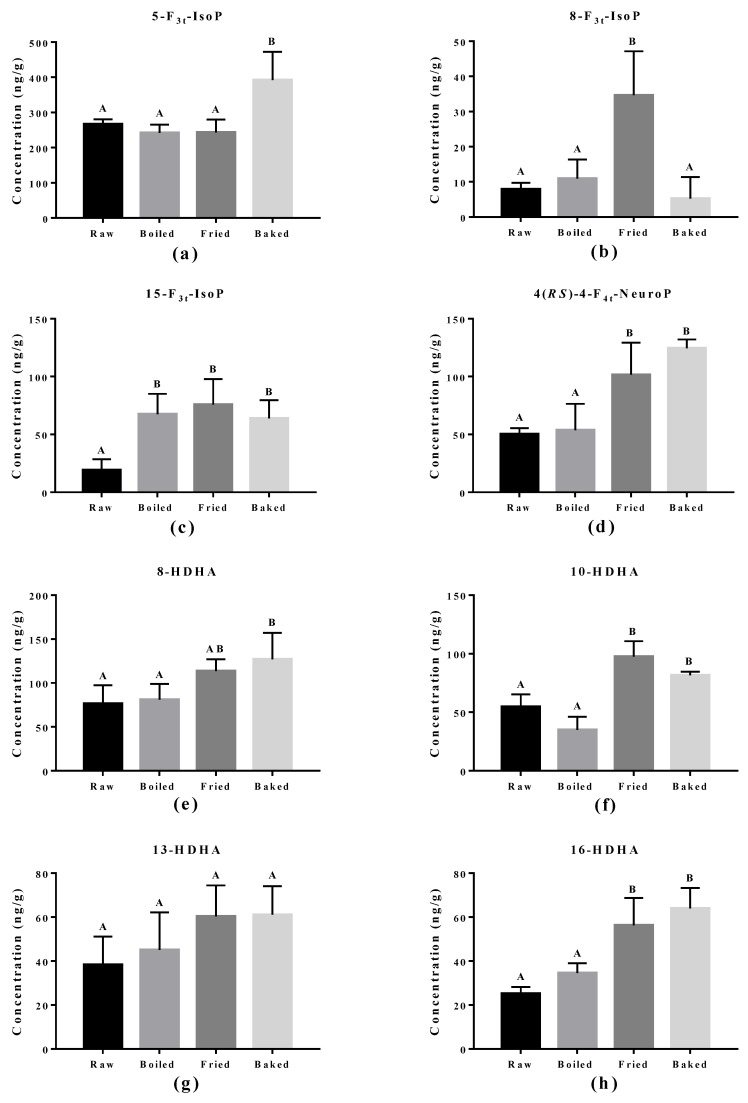
Concentration of non-enzymatically oxidized n-3 PUFA products measured in salmon samples. Graphs represent (**a**–**c**) F_3_-IsoPs derived from eicosapentaenoic acid and (**d**–**h**) NeuroPs and hydroxy-DHA (HDHA) derived from docosahexaenoic acid. Data are presented as the mean ± S.D. (*n* = 4). Raw: control salmons without any cooking; boiled: salmons cooked by boiling; fried: salmons cooked by pan-frying; baked: salmons cooked by oven-baking. Similar letters denote no statistical differences between samples, otherwise it is statistically significant at *p* < 0.05.

The effect of cooking methods on the level of non-enzymatic-oxidized lipid products was more obvious (Figure 5 and Figure 6). The cooking method that led to the strongest lipid peroxidation in salmon was pan-frying, where a significant increase in the amount of 15-F_2t_-IsoP derived from AA, 15-F_3t_-IsoP and 8-F_3t_-IsoP derived from EPA, and 10-HDHA and 16-HDHA derived from DHA were detected. On the other hand, oven-baking also increased the levels of certain oxidized lipid products including 15-F_3t_-IsoP derived from EPA and 4-(*RS*)-4-F_4t_-NeuroPs, 10-HDHA and 16-HDHA derived from DHA. However, boiling the salmon only increased the concentration of 15-F_3t_-IsoP.

## 4. Discussion

Regular consumption of long chain n-3 PUFAs, namely EPA and DHA, has been proposed to have health benefits such as improving blood flow and blood pressure, cardiovascular disease prevention and neurodegenerative disease protection [2,4,30]. In general, fatty fish is a rich source of EPA and DHA. American Heart Association recommended to the public to consume regular fatty fish at least twice a week to obtain the health benefits of EPA and DHA [31]. However, fish in most countries is consumed cooked and not raw; therefore, concerns about the method of cooking may lead to the reduction of n-3 PUFAs and the generation of potentially toxic oxidized lipid products due to the high heat. 

In this study, the content of PUFAs, oxidized PUFA products and level of lipid peroxidation in raw, boiled, pan-fried and oven-baked salmon were examined. None of the cooking methods was able to significantly reduce either n-3 or n-6 PUFAs in the salmon samples, which indicates that the quality of n-3 PUFAs in benefitting health was not altered after cooking. This matched the findings in the study of Bastias et al., where the fatty acid profile in salmon remained unchanged after cooking by four different methods [32]. In a similar study, cooking methods including boiling, frying and roasting of humpback salmon showed that only frying significantly reduced EPA and DHA [13]. The group suggested that the decreased PUFAs in the salmon may be caused by the longer frying time. The frying time used was 15-20 min, but in this study, it took 10 min; in comparison, the longer frying time may have raised the oxidation of PUFAs in the salmon and subsequent loss of PUFAs. Although in this study, cooking oil was not used in the frying process, the reduction of PUFAs in pan-fried salmon may occur if cooking oil is added during frying. Flaskerud et al. found that pan-frying trout in corn oil and canola oil had no effect on the level of PUFAs in the fish, but pan-frying with peanut oil and high oleic sunflower oil induced the reduction of EPA and of EPA and DHA, respectively [33]. This matched the findings from Gladyshev et al., who used sunflower oil to fry the salmon [13]. The decline of EPA and DHA is the result of the fat of fish filets transferring to the cooking oil when pan-frying in sunflower oil. Sioen et al. proposed that the fat of the fish is leached out during the frying process to the cooking oil, and the free radicals generated in the hot cooking oil in return oxidize the fat of the fish [34]. Thereafter, another similar study was conducted by Gladyshev et al. with four different fish species including sea trout, herring, rock sole and cod [35]. They concluded that the difference of EPA and DHA content in fish depends on the fish type and the use of cooking oil, and not the cooking methods. In their report, only the fried Norwegian trout showed a significant reduction of EPA and DHA compared to raw trout. Again, the loss of EPA and DHA in fried Norwegian trout was mainly due to the sunflower oil during frying. 

Heating PUFA will generate numerous types of oxidized products that may be advantageous and disadvantageous to human health. Unexpectedly, PUFA levels were not significantly reduced by the cooking methods, but the results of the PV test, TBARS test, quantification of 4-HHE and 4-HNE and the oxidized products showed alteration. The large standard deviation of the mean PUFAs particularly after frying or baking signified that cooking methods perhaps had an impact on the PUFA concentration. Of the salmon samples, frying and baking induced lipid peroxidation in the salmon where frying had the strongest effect among the three cooking methods. The difference observed in the level of lipid peroxidation in salmon cooked by the different methods is attributed to the heat temperature. Boiling is a mild cooking method, and the temperature generated through boiling was the lowest compared to other cooking methods where the maximum is 100 °C, i.e., the boiling point of water. Moreover, the cooking time was not long compared to baking since the heat transfer by boiling is very effective as the salmon is immersed into the hot water, allowing energy transfer to the food by constant collision of the food molecules through water convection [36]. The short cooking time and low temperature may explain the low level of peroxides and reactive aldehydes in boiled fish compared to raw fish. 

On the other hand, pan-frying transfers heat through conduction at a temperature between 190 °C and 210 °C. Even though the temperature used in pan-frying is twice that of boiling, the cooking time is similar to boiling, as the temperature can only transfer to the surface of the food through conduction, which then rapidly dehydrates the food surface and causes Maillard browning and flavor development [35]. The interior of the food can remain moist, and the temperature usually does not exceed 100 °C, as it is insulated by the outer surface and the fat of the food. However, the temperature of the salmon surface is highly exposed to free radicals of the oil released by thermal oxidation in the presence of oxygen, i.e., air and water. As a consequence, the lipid peroxidation caused by frying is strong compared to other methods, and as a result, the levels of peroxides and reactive aldehydes are high.

As for baking, the temperature used was 200 °C and the cooking time was the longest (15 min) since the heat transfer in baking is air convection and radiation, which is not as efficient as boiling and frying. The air in the oven is a thousand-times less dense than water, so the energy transfer by collisions of hot air molecules and food are low [36]. Therefore, it takes more time to heat up the food in the oven, and the inner temperature of the food is much lower than the surrounding temperature. Subsequently, the rate of lipid peroxidation by baking is less strong compared to pan-frying, although the temperature used is similar and the cooking time longer.

The elevation of lipid peroxidation in salmon fillet after frying was further supported by the oxidized PUFA products measured. One product, 15-F_2t_-IsoP derived from AA, is formed via ROS/free radicals non-enzymatically and is a renowned in vivo oxidative stress biomarker in vivo. It was also significantly induced in the fried salmon sample compared to boiling or baking. This observation indicates frying does increase the free radical production through thermal oxidation of the salmon fillet. Similar products from n-3 PUFA, namely 15-F_3t_-IsoP and 8-F_3t_-IsoP from EPA and 4(*RS*)-4-F_4t_-NeuroP from DHA, are also formed through ROS/free radicals in non-enzymatic oxidation. Elevation of 15-F_3t_-IsoP was also found in boiled and baked salmon and no change in 8-F_3t_-IsoP, which matched the PV and TBARS findings that the level of lipid peroxidation produced from boiling and baking is less strong compared to frying. It was surprising to discover that the baking and frying methods increased several DHA-derived oxidized products, but most notably 4(*RS*)-4-F_4t_-NeuroP. Recent studies found that 4(*RS*)-4-F_4t_-NeuroP is cardio-protective, containing an anti-arrhythmic factor that can repair the RyR2 ryanodine receptor in the heart and maintain calcium homeostasis [17,18,37]. Furthermore, 4(*RS*)-4-F_4t_-NeuroP improved heart variability, reduced pro-inflammatory cytokines and reduced thrombosis with improved blood flow [17]. 

In this study, pan-frying the salmon generated high levels of DHA-derived oxidized products. This is probably due to the strong heat contact in pan frying and potentially promoted secondary lipid peroxidation to generate aldehydes such as 4-HHE and 4-HNE that can cross-link with the protein [38] in the salmon. The elevation of 4-HHE in pan-fried salmon may not be disadvantageous to human health. Although 4-HHE is known to be neurotoxic, to increase during digestion of fatty fish and even to induce gut inflammation [39,40], a low concentration of 4-HHE was found to be cardioprotective by activating Nrf2 [21,22]. Furthermore, the results of the TBARS assay also showed pan-frying to be the only cooking method strong enough to increase the level of MDA in salmon. Both results support that the high temperature contact and heat conduction of the frying led to further degradation of the oxidized products of DHA. 

## 5. Conclusions

The results of this study provided a new insight into how cooking methods affect the composition of PUFAs and oxidized PUFA products in salmon. Prior to the study, we hypothesized that it was possible to reduce the amount of PUFAs and increase their oxidized products in salmon through common cooking methods since the high temperature used could oxidize PUFAs due to the numerous double bonds in the structure. However, the results were unexpected. While the quantified inherent margin of error of the PUFAs showed no reduction after cooking, oxidized products of PUFAs were clearly increased, suggesting that indeed a proportion of the n-3 PUFA, namely EPA and DHA, was modified and generated bioactive compounds such as 4(*RS*)-4-F_4t_-NeuroP. 

## Figures and Tables

**Table 1:** Concentration of enzymatically-oxidized products derived from n-3 and n-6 PUFAs measured in salmon samples.

	Raw	Boiled	Fried	Baked
**Enzymatic oxidized lipid products derived from AA**				
5(*S*)-HETE	134.12 ± 29.53 ^a^	150.46 ± 33.12 ^a^	265.91 ± 92.79 ^a^	201.24 ± 7.79 ^a^
8(*S*)-HETE	19.98 ± 8.02 ^a^	21.30 ± 2.45 ^a^	56.83 ± 35.47 ^a^	29.80 ± 4.81 ^a^
11(*S*)-HETE	32.15 ± 9.16 ^a^	30.73 ± 7.76 ^a^	81.76 ± 43.66 ^a^	55.31 ± 27.44 ^a^
12(*S*)-HETE	138.95 ± 50.97 ^a^	235.53 ± 198 ^a^	142.66 ± 86.35 ^a^	98.54 ± 42.05 ^a^
15(*S*)-HETE	8.71 ± 4.42 ^a^	9.62 ± 4.32 ^a^	6.71 ± 2.77 ^a^	7.90 ± 2.69 ^a^
**Enzymatic oxidized lipid products derived from EPA**				
RvE1	8.97 ± 2.94 ^a^	14.31 ± 2.84 ^a^	25.55 ± 3.86 ^b^	11.80 ± 3.41 ^a^
**Enzymatic oxidized lipid products derived from DHA**				
4-HDHA	63.36 ± 17.08 ^a^	51.03 ± 10.09 ^a^	78.24 ± 15.16 ^a^	88.54 ± 17.42 ^a^
7-HDHA	36.45 ± 6.32 ^a^	35.28 ± 7.88 ^a^	47.03 ± 11.73 ^a^	54.47 ± 16.39 ^a^
11-HDHA	38.21 ± 4.84 ^a^	33.86 ± 8.98 ^a^	55.02 ± 7.74 ^a^	48.91 ± 13.09 ^a^
14-HDHA	40.43 ± 3.42 ^a^	35.62 ± 10.38 ^a^	84.71 ± 22.08^b^	89.26 ± 13.17 ^b^
17-HDHA	391.58 ± 43.80 ^a^	539.14 ± 149.46 ^a^	489.99 ± 36.43 ^a^	790.34 ± 273.12 ^a^
RvD1	19.46 ± 8.50 ^a^	19.11 ± 2.90 ^a^	23.82 ± 3.81 ^a^	37.43 ± 12.13 ^a^
NPD1	11.41 ± 3.01 ^a^	15.44 ± 2.70 ^a^	24.68 ± 6.20 ^s^	11.93 ± 2.33 ^a^

Data are presented as the mean ± S.D. (n = 4), ng/g of tissue. Raw: control salmons without any cooking; boiled: salmons cooked by boiling; fried: salmons cooked by pan-frying; baked: salmons cooked by oven-baking. HETE: hydroxyeicosatetraenoic acid; HDHA: hydroxy-DHA; RvD1: resolving D1; NPD1: neuroprotection D1. Similar letters in superscript denote no statistical differences between samples, otherwise it is statistically significant at *p* < 0.05.
